# Deep Learning for Epileptic Seizure Detection Using a Causal-Spatio-Temporal Model Based on Transfer Entropy

**DOI:** 10.3390/e26100853

**Published:** 2024-10-10

**Authors:** Jie Sun, Jie Xiang, Yanqing Dong, Bin Wang, Mengni Zhou, Jiuhong Ma, Yan Niu

**Affiliations:** 1 College of Computer Science and Technology (College of Data Science), Taiyuan University of Technology, Taiyuan 030024, China; sunjie0086@link.tyut.edu.cn (J.S.); xiangjie@tyut.edu.cn (J.X.); d15110464375@163.com (Y.D.); wangbin01@tyut.edu.cn (B.W.); 2 School of Software, Taiyuan University of Technology, Taiyuan 030024, China; mn.zhou@siat.ac.cn; 3 Shanxi Provincial People’s Hospital, Taiyuan 030024, China; mjh2003@163.com

**Keywords:** epilepsy detection, transfer entropy, graph attention network, bi-directional long short-term memory network, spatiotemporal correlation

## Abstract

Drug-resistant epilepsy is frequent, persistent, and brings a heavy economic burden to patients and their families. Traditional epilepsy detection methods ignore the causal relationship of seizures and focus on a single time or spatial dimension, and the effect varies greatly in different patients. Therefore, it is necessary to research accurate automatic detection technology of epilepsy in different patients. We propose a causal-spatio-temporal graph attention network (CSTGAT), which uses transfer entropy (TE) to construct a causal graph between multiple channels, combining graph attention network (GAT) and bi-directional long short-term memory (BiLSTM) to capture temporal dynamic correlation and spatial topological structure information. The accuracy, specificity, and sensitivity of the SWEZ dataset were 97.24%, 97.92%, and 98.11%. The accuracy of the private dataset reached 98.55%. The effectiveness of each module was proven through ablation experiments and the impact of different network construction methods was compared. The experimental results indicate that the causal relationship network constructed by TE could accurately capture the information flow of epileptic seizures, and GAT and BiLSTM could capture spatiotemporal dynamic correlations. This model accurately captures causal relationships and spatiotemporal correlations on two datasets, and it overcomes the variability of epileptic seizures in different patients, which may contribute to clinical surgical planning.

## 1. Introduction

Epilepsy is a neurological disorder caused by a sudden abnormal discharge of brain neurons [1], with a global population of 70 million epilepsy patients [2]. If epileptic seizures are accurately detected, it can help medical staff develop corresponding surgical plans accurately. At present, the detection of epileptic seizures relies mainly on clinical doctors to identify epileptic seizures through visual analysis of electroencephalogram (EEG) special waveforms, which is time-consuming and inefficient. Therefore, there is an urgent need for computer-based automatic detection technology of epilepsy to accurately develop surgical plans.

Traditional machine learning methods are based on manual feature extraction, focusing only on single-dimensional information and classifying only a single channel or key channel, which undoubtedly results in the loss of important information. In recent years, an increasing number of researchers have incorporated deep learning algorithms such as convolutional neural networks (CNNs) [3,4] and long short-term memory (LSTM) [5,6] into medical diagnoses. In particular, compared with traditional machine learning algorithms, deep learning models have significant advantages in automatically extracting and selecting relevant features, making them very suitable for processing intracranial electroencephalogram (iEEG) signals with millisecond level temporal and spatial resolution [7]. Si XP et al. [8] proposed a new lightweight convolutional neural network model that combines convolutional block attention modules, achieving specificity of 85.4% on the SWEC dataset. Guo LH et al. [9] proposed a spatial dynamic graph convolutional network to construct spatial relationships between electrodes, with a sensitivity of 95%.

There are already deep learning models for detecting iEEG epilepsy data [10,11]. For example, Sun YL et al. [12] and others proposed an end-to-end model to evaluate long-term iEEG data, achieving a sensitivity of 97.5%. Wang, XS et al. [13] proposed a method of one-dimensional convolutional neural networks combined with a channel selection strategy for seizure prediction. For the iEEG dataset, a sensitivity of 90.09% was achieved at the segmentation level. Geng, DV et al. [7] used a long short-term memory network architecture and an auxiliary classifier generative adversarial network to train spike events in iEEG recordings of epilepsy patients. It is noted that these studies ignore the transmissibility of epileptic seizures.

Using graph neural networks for analysis can better consider the propagation process of neural electrical signals in the brain network during epileptic seizures, which is beneficial for the detection of epileptic seizures. Johnstone et al. [14] established a multidimensional enhancement measurement model using wavelet packet decomposition, CNN, and gated recurrent units, and achieved good results. Zhang YF et al. [15] constructed a global local graph convolutional neural network in a data-driven manner to learn the relevant graph structure and weights for intracranial electroencephalogram signals to optimize the learned feature representation and achieve the goal of epilepsy detection. Therefore, this article will focus on the detection of epileptic seizures based on deep learning as the primary research task.

The electrode distribution characteristics of intracranial electroencephalography provide a non-Euclidean topological structure. Therefore, representing it as a two-dimensional signal may result in a loss of information about the connectivity between functional brains. The study of electroencephalography based on graph theory analysis has been applied to different mental illnesses to explore the functional connectivity patterns of the brain [16,17,18]. There is certainly a causal relationship between electrodes during epileptic seizures, and transfer entropy (TE) can finely characterize the causal characteristics of information in brain regions [19]. It is more effective in identifying causal relationships and has fewer false detections, and TE has been widely used to analyze the interactions between brain regions during epileptic seizures [20,21,22]. Therefore, our paper uses TE to construct the causal relationship matrix of iEEG.

To address the above challenges, our paper proposes a causal-spatio-temporal graph attention network (CSTGAT) model that combines the directional information, temporal correlation of iEEG signals, and the spatial topology information of multi-channel electrodes, enabling epilepsy detection in different patients. The reliability of the model was demonstrated through ablation experiments and comparative experiments using two datasets. The experimental results indicate that the model performs well on both the private and SWEZ datasets and can be used for the detection of epilepsy.

## 2. Materials and Methods

### 2.1. CSTGAT Model

**Figure 1:**
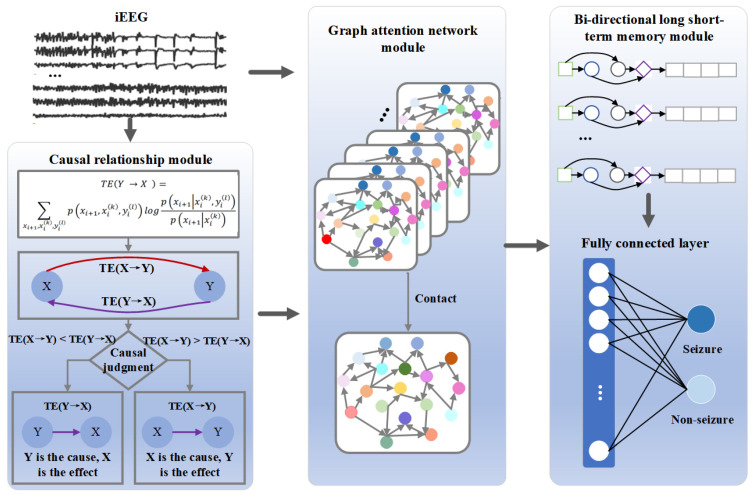
The framework of CSTGAT.

The structure of the CSTGAT model is shown in Figure 1. The model mainly consists of three modules: (1) Causal relationship module: this calculates the causal relationship between the original iEEG signals through TE and constructs a graph structure based on this causal matrix. The graph structure and the original signal are used as inputs for the model. (2) Graph attention network (GAT) module: The signal is input into the GAT model for spatial feature extraction. The GAT model assigns different weights to each node based on its importance and aggregates information from adjacent nodes accordingly. (3) Bi-directional long short-term memory (Bi-LSTM) module: The signal is input into Bi-LSTM to mine its time series characteristics and output data containing spatiotemporal and directional features. Finally, the softmax function is used for classification detection.

#### 2.1.1. Causal Relationship Module

We use the TE algorithm to identify the causal and dynamic information flow between time series, and we construct a causal relationship graph as input for the GAT model’s graph structure. We chose TE mainly because it can accurately capture causal relationships in complex network processes. Specifically, based on transmission probability, TE naturally integrates directional and dynamic information, and can finely characterize the orientation and dynamic information transmission of brain regions [23]. It is effective in identifying causal relationships and able to reliably observe nonlinear interactions, thereby reducing false detections. Moreover, this method does not require prior knowledge and can still detect causal connections in the presence of delays [24,25].

A brief overview of the TE method is as follows [19,21,26]:

${\left\{x_{i}\right\}}_{i=1}^{N}$ and ${\left\{y\right\}}_{i=1}^{N}$ are time series of 2 systems, X and Y. Using information entropy to study the degree of information transfer between variables

(1) $$\begin{array}{c} H_{X}=-\sum_{i}P\left(x_{i}\right)\log P\left(x_{i}\right) \end{array}$$

Each observation $x_{i}$ is regarded as a state of a Markov process and satisfies

(2) $$\begin{array}{c} p\left(x_{i+1}|x_{i},\ldots ,x_{i-k+1}\right)=p\left(x_{i+1}|x_{i},\ldots ,x_{i-k}\right) \end{array}$$

Taking into account all previous states, the average information transmission of $x_{i+1}$ can be obtained as follows:

(3) $$\begin{array}{c} h_{x}=\\ -\sum_{i}p\left(x_{i+1}|x_{i},\ldots ,x_{i-k+1}\right)\log p\left(x_{i+1}|x_{i},\ldots ,x_{i-k+1}\right) \end{array}$$

Therefore, the influence of Y on X can be calculated by computing the probability difference between the 2 systems, and the TE is defined as

(4) $$TE\left(Y\to X\right)=\sum_{x_{i+1},x_{i}^{\left(k\right)},y_{i}^{\left(l\right)}}p\left(x_{i+1},x_{i}^{\left(k\right)},y_{i}^{\left(l\right)}\right)log\frac{p\left(x_{i+1}|x_{i}^{\left(k\right)},y_{i}^{\left(l\right)}\right)}{p\left(x_{i+1}|x_{i}^{\left(k\right)}\right)}$$

where $x_{i}^{k}=p\left(x_{i+1}|x_{i},\ldots ,x_{i-k+1}\right)$ and $y_{i}^{l}=p\left(x_{i+1}|x_{i},\ldots ,x_{i-k+1},y_{i},\ldots ,y_{i-l+1}\right)$. The transfer entropy from Y to X is essentially the information that Y changes due to the uncertainty in X; that is, the amount of information transmitted from Y to X [27,28]. Therefore, by using TE, a causal relationship matrix can be constructed.

#### 2.1.2. Graph Attention Network Module

Using the causal relationship matrix constructed by TE and the iEEG segments as inputs, spatial features are extracted through the GAT model. Different weights are assigned to each node based on its importance and information is aggregated from neighboring nodes accordingly.

GAT is a special neural network designed specifically for processing graph structured data; it fully considers the relationships between data when processing input data and automatically learns the relationships between nodes [29,30,31]. The GAT model is based on an attention mechanism, which dynamically adjusts the connection weights between different nodes and adaptively aggregates the information of neighboring nodes, thus achieving more efficient, flexible, and accurate modeling on graph structured data [32,33]. Compared to traditional graph neural network models, GAT has the following advantages: firstly, it can adaptively learn the feature importance of each node and more accurately express the interrelationships between nodes; secondly, it can effectively alleviate the sparsity and noise problems in graphic data and better handle large-scale graphic data [34,35].

**Figure 2:**
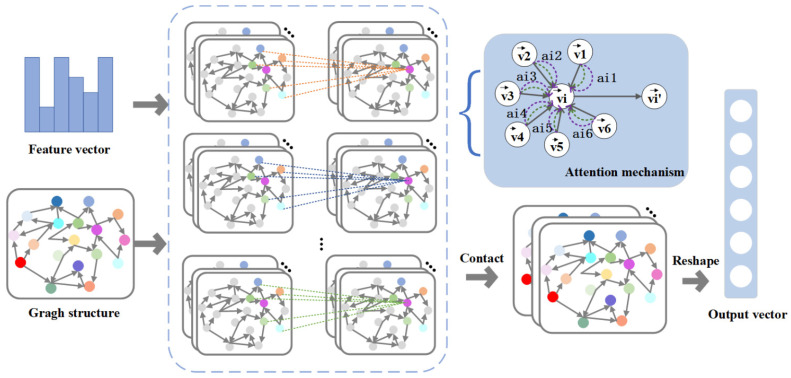
Graph attention network.

As shown in Figure 2, we input the node features and causal relationship matrix. Each layer aggregates nodes and their adjacent nodes based on the weight size; we then normalize and introduce a multi-head attention mechanism. Finally, the fused features are outputted.

Specifically, first take the node features $V=\{v_{1},v_{2},\ldots ,v_{N}\}$, where $v_{i}\in R^{F}$, and the causal relationship matrix at time t as inputs for the GAT model. Among them, N represents the number of electrode channels and F represents the feature size of each node. In the GAT layer, the self-attention mechanism is applied to each node in the graph, assigning different weights based on their importance. Subsequently, the layer aggregates nodes and their neighboring nodes based on the weight size. For a pair of nodes with input feature vectors $v_{i}$ and $v_{j}$, the influence of node i on node j is represented as $e_{ij}$, as follows:

(5) $$\begin{array}{c} e_{ij}=a\left(Wv_{i},Wv_{j}\right) \end{array}$$

The weight matrix W is used to establish the relationship between the input and output features, thereby achieving the transformation of node features. The feedforward neural network a(·) maps features to real numbers. This formula calculates the significance of nodes j to i without considering the structural information of the graph. To merge the correlation coefficients between nodes and their first-order neighborhoods, the calculation of $e_{ij}$ is limited to node $j\in G_{i}$, where $G_{i}$ represents the neighborhood of node i. Next, apply the softmax function to normalize the weights of all adjacent nodes as follows:

(6) $$\begin{array}{c} \alpha_{ij}={softmax}_{j}\left(e_{ij}\right)=\frac{\exp\left(e_{ij}\right)}{\sum_{l\in V_{i}}\exp\left(e_{il}\right)} \end{array}$$

The attention coefficient $\alpha_{ij}$ represents the attention between node i and node j, where l represents a node in the first-order neighbor set of i. By introducing the LeakyReLU activation function and extending the equation, the attention coefficient calculated by the attention mechanism can be expressed as follows:

(7) $$\begin{array}{c} \alpha_{ij}=\frac{\exp\left(\mathrm{LeakyReLU}\left(a^{T}\left[Wv_{i}∥Wv_{j}\right]\right)\right)}{\sum_{l\in G_{i}}\exp\left(\mathrm{LeakyReLU}\left(a^{T}\left[Wv_{i}∥Wv_{l}\right]\right)\right)} \end{array}$$

where ∥ represents the contact operation.

The ability of the single-layer attention mechanism to learn from adjacent nodes is relatively limited. To improve the accuracy of learning features, our study combines multiple attention mechanisms. By using a multi-head attention mechanism to calculate the attention coefficients of surrounding nodes, the learning process of the model becomes more stable. The fused feature output obtained, represented as $v_{i}^{\prime }$, can be expressed as follows:

(8) $$\begin{array}{c} v_{i}^{\prime }=\sigma \left(\frac{1}{k}\sum_{k=1}^{k}\sum_{j\in G_{i}}\alpha_{ij}^{k}W^{k}v_{j}\right) \end{array}$$

where k represents the head, $W^{k}$ is the weight matrix of k, σ is the activation function, and $v^{\prime }$ represents the output of the GAT model.

The attention mechanism in this model learns and parameterizes the connections between nodes, allowing for different weights to be assigned to each edge. In addition, each head in the multi-head attention mechanism operates independently and in parallel, eliminating the need for complex matrix operations such as eigenvalue decomposition. By introducing the attention mechanism, weights are only shared with adjacent nodes, eliminating the need for the entire graph information. This robustness enables the model to effectively handle interrupts. If the connection between 2 nodes is lost, their attention coefficients will not be calculated. Therefore, the model effectively extracts spatial relationships between channels [36].

#### 2.1.3. Bi-LSTM Module

Spatial features from the GAT module are extracted and input into the Bi-LSTM module to capture temporal relationships, ultimately outputting data containing spatiotemporal and causal features, and classifying the signals.

A long short-term memory network (LSTM) is a recurrent neural network (RNN) that solves the gradient explosion problem in traditional RNNs by introducing gating mechanisms [37,38]. The key components of an LSTM are cell states and various gating mechanisms, including forget gate $f_{t}$, input gate $i_{t}$, and output gate $o_{t}$, which control the degree of information flow through the sigmoid activation function. For a given time step t and LSTM, the operation is as follows:

(9) $$\begin{array}{c} f_{t}=\sigma \left(W_{f,m}m_{t}+W_{f,h}h_{t-1}+b_{f}\right) \end{array}$$

(10) $$\begin{array}{c} i_{t}=\sigma \left(W_{i,m}m_{t}+W_{i,h}h_{t-1}+b_{i}\right) \end{array}$$

(11) $$\begin{array}{c} \tilde{c_{t}}=\tanh\left(W_{\tilde{c},m}m_{t}+W_{\tilde{c},h}h_{t-1}+b_{\tilde{c}}\right) \end{array}$$

(12) $$\begin{array}{c} o_{t}=\sigma \left(W_{o,m}m_{t}+W_{o,h}h_{t-1}+b_{o}\right) \end{array}$$

(13) $$\begin{array}{c} c_{t}=f_{t}⊙c_{t-1}+i_{t}⊙\tilde{c_{t}} \end{array}$$

(14) $$\begin{array}{c} h_{t}=o_{t}⊙\tanh\left(c_{t}\right) \end{array}$$

Here, $m_{t}$ represents the input vector, b represents the bias term, $c_{t}$ represents the cell state, $\tilde{c_{t}}$ represents the candidate value vector, $h_{t}$ represents the output vector, and W represents the weight matrix.

The LSTM does not consider future information and only processes past information. The detection of intracranial electroencephalography requires comprehensive consideration of contextual information; therefore, our model adopts BiLSTM. BiLSTM calculates the hidden states ${\vec{h}}_{t+1}$ and ${\overset{\leftarrow }{h}}_{t+1}$ by running the LSTM layer forward and backward along the time axis, and then connects the 2 hidden states to form the final bi-directional hidden state.

### 2.2. Evaluation Indicators

The effectiveness of detection is evaluated using accuracy (Acc), sensitivity (Sen), specificity (Spe), precision (Pre), and F1-Score. Accuracy provides an overall evaluation of classification performance, while sensitivity and specificity are used to assess the model’s ability to correctly identify pre-seizure and inter-seizure data, thereby evaluating the accuracy of seizure detection. The formulae are as follows:

(15) $$\begin{array}{c} Acc=\frac{TP+TN}{TP+FP+TN+FN} \end{array}$$

(16) $$\begin{array}{c} Sen=\frac{TP}{TP+FN} \end{array}$$

(17) $$\begin{array}{c} Spe=\frac{TN}{TN+FP} \end{array}$$

(18) $$\begin{array}{c} Precision=\frac{TP}{TP+FP} \end{array}$$

(19) $$\begin{array}{c} F1-score=\frac{2\times Sen\times Precision}{Sen+Precision} \end{array}$$

### 2.3. Experimental Environment and Parameters

The experimental environment is the Windows 10 operating system, with Python 3.11.6 as the programming language and Pytorch as the deep learning framework (version 2023.1).

In the model training of this experiment, Epoch = 500. Cross entropy was used as the loss function with Adam as the optimizer. The relevant parameters of the GAT model are set as follows: number of heads = 8, learning rate = 0.0005, weight decay = 1 × 10^−5^, and dropout rate = 0.2. The hidden layer of BiLSTM is set to 192.

### 2.4. Datasets

We have used 2 datasets: (1) The SWEZ dataset, consisting of 16 drug-resistant epilepsy patients who underwent an epilepsy surgery evaluation at the Sleep Awakening Epilepsy Center (SWEC) of the University Neurology Department at Bern Hospital. The iEEG records EEG signals through strip, grid, and depth electrodes. After 16-bit analog-to-digital conversion, the data are digitally bandpass filtered using a fourth-order Butterworth filter between 0.5 and 150 Hz before analysis, and are written to the disk at a rate of 512 Hz. All EEG recordings are visually examined by experienced epileptic experts certified by the medical committee. These recordings are used to identify epileptic seizures and their resolution and exclude channels that are continuously disrupted by artifacts.

(2) The SPE dataset: iEEG data were collected from 6 patients in the neurosurgery department of Shanxi Provincial People’s Hospital. Trained epilepsy specialists strictly screen epilepsy patients according to inclusion and exclusion criteria, and the data collection process follows the principles and standard requirements for EEG signal recording proposed by the International Psychophysiological Society. The number of electrodes per patient ranges from 6 to 15 (average = 10) and the total number of contacts ranges from 99 to 194. The sampling rate is 512 Hz. The ROSA robot is used for electrode implantation, with the following parameter settings: diameter, 0.8 mm; length, 2 mm; spacing, 1.5 mm.

The pre-seizure period allows doctors sufficient time for clinical intervention, with the optimal duration typically being 3–5 min. In addition, the duration of epileptic seizures is comparable to the pre-seizure period. When setting the duration of the seizure period, balance must be maintained to ensure that it is not too long, otherwise it may bring psychological pressure to the subjects. According to the relevant literature, we set the pre-seizure period to 5 min and the seizure period to 30 min.

We use Brainstorm to pre-process the data. The frequency is uniformly 512 Hz. To overcome the data imbalance between the pre-seizure and seizure periods, we use overlapping sliding windows to extract pre-seizure segments, ensuring a fairer proportion of training data between the 2 classes. By utilizing a sliding window analysis, we can effectively divide long-distance iEEG signals into different segments, ensuring a sufficient number of samples for training deep neural networks.

## 3. Results and Discussion

### 3.1. Subject-Specific Experiments

The performance of the GSTAT model is evaluated through multi-patient experiments on the iEEG datasets of SWEZ and SPE. As shown in Table 1, the accuracy of patient ID11 and ID13 in this model is the highest in the public dataset, reaching 99.68% and 99.45%, respectively. Patients ID01, ID03, ID05, ID06, ID08, ID09, ID10, ID15 and ID16 also have relatively high accuracy rates of 99.05%, 98.98%, 99.08%, 99.31%, 98.93%, 98.83%, 99.2%, 98.88% and 98.9%, respectively, all of which are around 99%. The accuracy of patients ID02, ID12, ID14, and ID16 around 98%. The accuracy rates of patient ID04 and ID07 are relatively low, at 95.79% and 97.28%, respectively. The specificity of patient ID04 is relatively low, only 94.66%, but compared with existing studies, there has been an improvement. The model performs well in private datasets, with an accuracy of over 98.5% and high specificity and sensitivity (Table 1).

Some patients have poor recognition performance, which may be due to severe epileptic seizures and interference in signal acquisition. It is also possible that due to the large differences between patients, the duration of attacks and the involved areas may vary, and poor generalization of the model can lead to unsatisfactory performance. The CSTGAT model proposed in our study performs well in both public and private datasets, and the model can effectively detect epileptic seizures in different patients with good robustness.

### 3.2. Ablation Experiments

**Figure 3:**
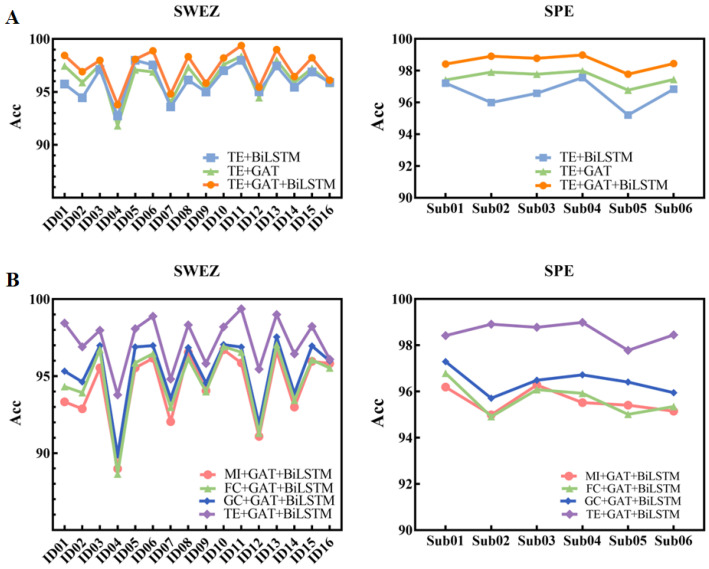
Ablation experiment results of different patients. (**A**) the performance of the components; (**B**) the performance of different network construction methods.

To demonstrate the performance of the components, different patient ablation experiments were conducted on the iEEG datasets of SWEZ and SPE. As shown in Figure 3A, the CSTGAT model (orange) showed some improvement compared to the TE+GAT model (green) and the TE+BiLSTM model (blue) in different patients. As shown in Table 2, the average accuracy of public datasets ultimately reached 97.24%, while the average accuracy of private datasets ultimately reached 98.55%. This proves the necessity of the GAT component and the BiLSTM component, indicating that these two components can accurately extract spatiotemporal features.

Furthermore, the performance of different network construction methods, such as granger causality (GC), functional connection (FC), and mutual information (MI) methods, was compared. The GC method determines whether there is a causal relationship between Y and X. FC can be understood as the interdependence of existing uncertainties. MI can be seen as the amount of information contained in one random variable about another random variable. As shown in Figure 3B, among different patients, the CSTGAT model, which uses TE as the network-constructing method (purple), achieved the highest accuracy. Thereafter, the accuracy from high to low is ordered as GC (blue), FC (green), and MI (orange). As shown in Table 2, the average accuracy of public datasets is at a low of 94.38% (MI+GAT+BiLSTM) and high of 97.24% (TE+GAT+BiLSTM), while the accuracy of private datasets is at a low of 95.59% (MI+GAT+BiLSTM) and at a high of 98.55% (TE+GAT+BiLSTM). This proves that the causal relationship matrix extracted by TE is more effective at capturing information than other methods.

### 3.3. Comparison with Other Methods

For the same public datasets, the comparison between our model and existing research [39] is shown in Table 3, and the average accuracy has increased from 95.42% to 97.24%. In the four patients with poor performance in previous studies, there is also a certain degree of improvement (only sensitivity and specificity were reported for different patients in the original text). As shown in Table 4, the specificity of patient ID04 has increased from 79.97% to 89.28%, and the accuracy reached 93.79%. Similarly, the sensitivity of patient ID05 has increased from 80% to 98.99%, with an accuracy rate of 98.08%; the sensitivity of patient ID12 has increased from 85.71% to 92.45%, with an accuracy rate of 95.45%; and the specificity of patient ID14 has increased from 49.9% to 98.24%, with an accuracy rate of 96.45%. Compared to existing research, the CSTGAT model proposed in this article performs well in various indicators and special patients, demonstrating excellent performance.

Due to the limited research on detection in public datasets of the same batch, this paper added comparative experiments with traditional graph convolutional networks (GCN). The input terminals are all causal relationship matrices and EEG segments, and the performance of different graph convolution models is compared. In Table 4, it can be observed that the accuracy of the traditional GCN model is only 79.33%, which may be because the model updates nodes by averaging or summing the features of neighboring nodes, ignoring the importance differences between neighboring nodes. In contrast, GAT introduces attention mechanisms that allow the model to learn the importance weights of each neighboring node to the current node, resulting in significant performance improvements in graph structured data processing.

### 3.4. Influence of the Parameters

**Figure 4:**
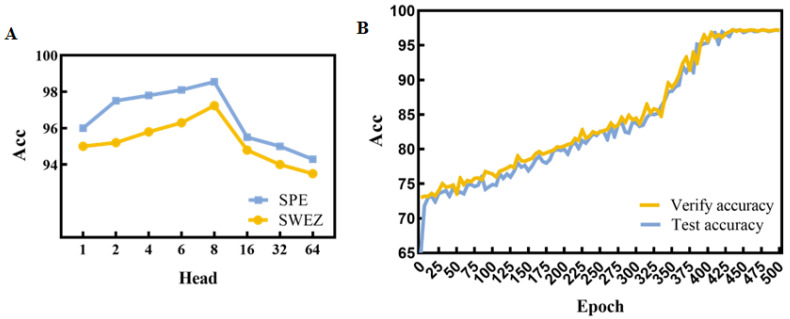
Influence of the parameters. (**A**) comparison of the experimental results with a different number of heads; (**B**) variation in accuracy with epochs.

The number of heads in the CSTGAT model is a critical parameter that represents the learning ability of multiple sub-representation layers. Too few subspaces can result in insufficient feature extraction, while too many heads can lead to overrepresentation and extraction of redundant information. Therefore, we conducted comparative experiments on the impact of the number of heads in the GAT network layer on model performance. The experimental results, as shown in Figure 4A, achieved high accuracy for the eight heads in both datasets. Therefore, this paper uses an eight-head attention network. Figure 4B presents the variation of accuracy with epochs, and it can be observed that the model tends to converge after approximately 435 epochs.

The CSTGAT model is an epilepsy detection method for different patients that combines the directional information and temporal correlation of iEEG signals with the spatial topology information of multi-channel electrodes. The main advantages of this method are as follows: the use of TE can accurately extract spatial information and causal relationship information between iEEG signal channels; temporal dynamic information can be accurately captured through GAT; and BiLSTM can be used for implementing epileptic seizure detection tasks. The robustness and generalization of CSTGAT have been demonstrated through different patient detection experiments on public and private datasets. Conducting ablation experiments on key modules has demonstrated the necessity of each module. Comparative experiments have also shown that the model is superior on average and for specific patients. Future work can improve GAT by adding models such as Transformer for refinement and by changing the input end and trying a new causal network building method.

## 4. Limitations

In future work, it is necessary to expand the size of the intracranial epilepsy dataset and collect more patient data to improve the model. Although the attention mechanism in this model can automatically learn key information from EEG data and understand its spatiotemporal features, there are still some limitations in fully understanding its classification mechanism. Therefore, it is necessary to further introduce interpretable learning methods to meet the clinical requirements for model interpretability. In epilepsy detection technology, determination of the lesion location is also a noteworthy issue, and further improvement of the model to assist clinical localization is needed in future work. In the causal relationship module, we can try the improved version of TE to explore whether the accuracy will be further improved.

## 5. Conclusions

We propose a graph spatiotemporal attention network based on a causal relationship, which constructs causal relationships between multiple channels through TE and combines a graph attention network and bi-directional long short-term memory network to capture spatiotemporal information. The robustness and generalization of CSTGAT have been demonstrated through different patient detection experiments on public and private datasets. Further ablation experiments were conducted on key components to demonstrate the necessity of each component. Finally, compared with existing studies, the average level and performance of this model on special patients are better. In summary, this model can effectively detect epilepsy in different patients using multi-channel iEEG, and it can be extended to the design of clinical decision-making systems.

## Figures and Tables

**Table 1:** Subject-specific experiments using the two datasets, SWEZ and SPE.

Dataset	Patients	Acc	Sen	Spe	F1-Score
SWEZ	ID01	99.05	98.95	99.88	98.93
	ID02	97.91	98.83	99.92	98.72
	ID03	98.98	100	100	99.58
	ID04	95.79	95.99	94.66	93.60
	ID05	99.08	99.58	100	98.79
	ID06	99.31	100	99.05	97.18
	ID07	97.28	97.88	98.46	97.87
	ID08	98.93	100	99.88	99.62
	ID09	98.83	98.64	99.72	98.62
	ID10	99.2	99.65	99.67	99.11
	ID11	99.68	100	100	99.72
	ID12	98.45	97.29	98.77	95.95
	ID13	99.45	100	100	99.75
	ID14	98.25	97.88	98.99	96.45
	ID15	98.88	99.68	99.83	98.32
	ID16	98.9	98.35	97.59	96.70
	Average	98.64	98.90	99.18	98.09
SPE	Sub01	99.02	100	99.92	99.45
	Sub02	99.59	99.76	98.95	99.19
	Sub03	99.58	98.99	99.72	99.33
	Sub04	99.69	100	100	99.16
	Sub05	98.98	100	98.97	97.89
	Sub06	99.25	98.88	100	98.83
	Average	99.35	99.61	99.59	98.98

**Table 2:** Ablation experiment results of two datasets.

Dataset	Model	Acc	Sen	Spe	Dataset	Model	Acc	Sen	Spe
SWEZ	TE+GAT	96.28	97.37	97.54	SPE	TE+GAT	97.56	97.86	98.65
	TE+BiLSTM	96.00	97.09	96.99		TE+BiLSTM	96.57	96.88	98.25
	MI+GAT+BiLSTM	94.38	95.66	96.46		MI+GAT+BiLSTM	95.59	96.48	96.99
	FC+GAT+BiLSTM	94.75	95.06	96.84		FC+GAT+BiLSTM	95.67	97.06	96.85
	GC+GAT+BiLSTM	95.37	96.25	97.73		GC+GAT+BiLSTM	96.43	97.89	97.25
	TE+GAT+BiLSTM	97.24	97.92	98.11		TE+GAT+BiLSTM	98.55	99.06	99.15

**Table 3:** Comparison of the experimental results.

Model	Acc	Sen	Spe
Burrello et al. [39]	95.42	96.01	94.84
GCN	79.33	78.64	89.92
GAT	96.28	97.37	97.54
BiLSTM	96.00	97.09	96.99
GCN+BiLSTM	85.62	87.25	92.58
GAT+BiLSTM	97.24	97.92	98.11

**Table 4:** Comparison of the subject-specific experiments.

Patients	CSTGAT			Burrello et al. [39]		
	Acc	Sen	Spe	Acc	Sen	Spe
ID04	93.79	94.02	89.28	NA	91.03	79.97
ID05	98.08	98.99	100	NA	80	96.88
ID12	95.45	92.45	96.45	NA	85.71	95.94
ID14	96.45	97.67	98.24	NA	88.57	49.9

## Data Availability

The SWEZ dataset can be found on the website (http://ieeg-swez.ethz.ch/, accessed on 15 January 2024). The causal relationship network building uses Matlab 2022, while the deep learning uses Python 3.11.6. Anyone with code requirements can contact the corresponding author.
